# INCT UroGen and the Transformation of Brazilian Urologic Oncology: A Model for Translational Science and Health Equity

**DOI:** 10.1590/S1677-5538.IBJU.2025.9914

**Published:** 2025-07-20

**Authors:** Leonardo O. Reis, Quoc-Dien Trinh, Shahrokh Shariat, Roger Chamas, Antonio C. R. Vallinoto

**Affiliations:** 1 Pontifícia Universidade Católica de Campinas Instituto de Imuno-Oncologia Campinas SP Brasil Instituto de Imuno-Oncologia, Pontifícia Universidade Católica de Campinas - PUC-Campinas, Campinas, SP, Brasil;; 2 Universidade Estadual de Campinas UroScience, Faculdade de Ciências Médicas Campinas SP Brasil UroScience, Faculdade de Ciências Médicas, Universidade Estadual de Campinas - Unicamp, Campinas, SP, Brasil;; 3 Instituto Nacional de Ciência, Tecnologia e Inovação INCT UroGen INCT em Câncer Geniturinário Campinas SP Brasil INCT UroGen, Instituto Nacional de Ciência, Tecnologia e Inovação - INCT em Câncer Geniturinário, Campinas, SP, Brasil;; 4 University of Pittsburgh School of Medicine Department of Urology Pittsburgh PA USA Department of Urology, University of Pittsburgh School of Medicine, Pittsburgh, PA, USA;; 5 Medical University of Vienna Department of Urology Comprehensive Cancer Center 1090 Vienna Austria Department of Urology, Comprehensive Cancer Center, Medical University of Vienna, 1090 Vienna, Austria;; 6 Medical University of Vienna Center for Cancer Research Vienna Austria Center for Cancer Research, Medical University of Vienna, Vienna, Austria;; 7 Karl Landsteiner Institute of Urology and Andrology Vienna Austria Karl Landsteiner Institute of Urology and Andrology, Vienna, Austria;; 8 Charles University Department of Urology Second Faculty of Medicine Prague Czech Republic Department of Urology, Second Faculty of Medicine, Charles University, Prague, Czech Republic;; 9 University of Jordan Department of Special Surgery Division of Urology Amman Jordan Division of Urology, Department of Special Surgery, University of Jordan, Amman, Jordan;; 10 Weill Cornell Medical College Department of Urology New York NY USA Department of Urology, Weill Cornell Medical College, New York, NY, USA;; 11 University of Texas Southwestern Department of Urology Dallas TX USA Department of Urology, University of Texas Southwestern, Dallas, TX, USA;; 12 Tabriz University of Medical Sciences Urology Department Research Centre for Evidence Medicine Tabriz Iran Research Centre for Evidence Medicine, Urology Department, Tabriz University of Medical Sciences, Tabriz, Iran;; 13 Faculdade de Medicina da Universidade de São Paulo Centro de Pesquisa Translacional em Oncologia Instituto do Câncer do Estado de São Paulo São Paulo SP Brasil Centro de Pesquisa Translacional em Oncologia, Instituto do Câncer do Estado de São Paulo, Faculdade de Medicina da Universidade de São Paulo - FMUSP, São Paulo, SP, Brasil;; 14 Universidade de São Paulo Centro Integrado de Oncologia de Precisão São Paulo SP Brasil Centro Integrado de Oncologia de Precisão, Universidade de São Paulo - USP, São Paulo, SP, Brasil;; 15 Universidade Federal do Pará Instituto de Ciências Biológicas Laboratório de Virologia Belém PA Brasil Laboratório de Virologia, Instituto de Ciências Biológicas, Universidade Federal do Pará - UFPA, Belém, PA, Brasil

## COMMENT

There is growing momentum in Brazilian urology, driven by the rise of impactful initiatives that are reshaping the national and international landscape. Chief among them is the International Brazilian Journal of Urology (IBJU), which has achieved its highest-ever Impact Factor: 4.5, according to the 2025 Journal Citation Reports. This milestone, up from 3.7 in 2023 and ~1.0 in 2019, secures IBJU's place among the top 10 urology journals worldwide (1).

Concurrently, Brazil launched its first national translational and clinical research flagship in urology and genitourinary oncology. The National Institute of Science, Technology and Innovation in Genitourinary Cancer (INCT UroGen) is a sister ecosystem of the "UroScience: Science Beyond Borders" initiative and forms a core pillar of the Navis Mater "Immuno-Oncology Institute", a multidisciplinary, innovation-driven platform anchored at the Pontifical Catholic University of Campinas (PUC-Campinas), and associated with the newly established Comprehensive Center for Precision Oncology at the University of São Paulo (https://c2po.usp.br/).

Supported by a significant federal investment of R$10.5 million (~US$2 million) from CNPq (National Council for Scientific and Technological Development), FAPESP (São Paulo Research Foundation), and the Brazilian Ministry of Health, INCT UroGen is headquartered at PUC-Campinas and its network spans all five Brazilian macroregions, forging an unprecedented alliance among leading universities, research centers, public hospitals, and regional stakeholders. INCT UroGen drives a comprehensive translational pipeline designed to inform national evidence-based guidelines and strengthen public health decision-making.

By embedding its scientific output within the infrastructure of the Brazilian Unified Health System (SUS), INCT UroGen is uniquely positioned to advance both scientific excellence and health equity, helping to bridge longstanding disparities in cancer care access across Brazil (2). The institute is closely aligned with the principles and trajectory of SUS, Brazil's globally recognized universal health system established in 1988. SUS is not only the backbone of public health delivery, but also a critical engine for research, surveillance, and the development of human capital (5).

To support the INCT UroGen in the SUS context, the PUC-Campinas has secured additional funding of over R$5.7 million (~US$1 million) through its "Immuno-Oncology Institute", leading a groundbreaking project in Brazil to develop a personalized vaccine against non-muscle invasive bladder cancer funded by Finep (Brazilian Funding Authority for Studies and Projects), through the "More Health Innovation" program.

Leveraging the SUS as a platform for innovation, INCT UroGen enhances the system's capacity to translate scientific knowledge into equitable, accessible care. This includes the validation of biomarkers and health technologies for cost-effectiveness, upholding ethical and equity-based principles, and reducing delays in diagnosis and treatment. In doing so, INCT UroGen helps build a more just and responsive health system where scientific progress directly serves public need.

Indeed, Brazil is championing a broader vision of research impact—one that moves beyond citations to encompass societal relevance, policy integration, and tangible public health outcomes. Social Impact Factor (SIF) frameworks are increasingly used to evaluate how research influences clinical guidelines, shapes health legislation, or transforms patterns of care within systems like SUS (6,7).

Long-term scientific sustainability of this initiative demands a diversified funding architecture, in which Brazil's research funders, CNPq, FINEP, and CAPES (Coordination for the Improvement of Higher Education Personnel) play distinct, complementary, strategic, and catalytic roles in advancing health science in Brazil. INCT UroGen exemplifies how structured, transparent, and mission-driven science can effectively attract such multilateral support, engaging the private sector, philanthropic foundations, and international cooperation frameworks, increasingly essential to scale innovation, expand infrastructure, and promote institutional resilience. The establishment of shared biobanks, interoperable research databases, and co-developed translational roadmaps not only strengthens scientific productivity but also boosts donor confidence and cross-sector accountability.

Together, the Brazilian Ministry of Education, the Ministry of Health, and the Ministry of Science, Technology, and Innovation help ensure Brazil's health science ecosystem is both academically robust and innovation-driven, linking education, research, and real-world health solutions through its agencies. CNPq fosters a strong national research base and drives the generation of evidence that informs public health policies, clinical guidelines, and innovation within the SUS (8). CAPES is central to human capital development, funding graduate programs, scholarships for Master's and PhD students, and international exchange (9). Finep supports research institutions, technological development, and public-private partnerships, focusing on health technology innovation and infrastructure, funding laboratories and equipment (10).

It has been instrumental in enabling multidisciplinary consortia like INCT UroGen to emerge and thrive. Through investments in thematic projects, young investigator grants, and institutional infrastructure, the INCT UroGen amplifies national impact, enabling the institute to establish its headquarters in São Paulo while expanding collaborative nodes across all five Brazilian macroregions. As one of Latin America's most respected public funding agencies, FAPESP is known for its rigorous peer review, long-term programmatic funding, and global outlook, positioning São Paulo and Brazil more broadly as a leader in internationally visible, socially relevant science, encouraging science-industry synergy, and accelerating the real-world application of research outputs (11).

These frameworks enable Brazilian researchers to co-lead global projects, share data and infrastructure, and raise Brazil's visibility within the international scientific community. In the INCT UroGen initiative, Brazilian Government Funding Agencies’ integration and influence extend beyond funding—it provides strategic coherence, institutional legitimacy, and a long-term vision.

As a global plan of action, international collaboration is not ancillary; it is a core strategic axis of INCT UroGen's mission. Through active alignment with premier cancer centers, the institute accelerates knowledge exchange, joint discovery, and translational co-development, maintaining strategic agreements with international funding bodies such as the NIH (USA), CNRS (France), DFG (Germany), and Horizon Europe (EU).

These international partnerships allow Brazil not only to contribute meaningfully to global cancer research but also to help fill critical scientific gaps, particularly in underrepresented populations and low- to middle-income settings. INCT UroGen fosters both South-to-South and South-to-North scientific pipelines, ensuring a more balanced and inclusive global research ecosystem. These collaborations are underpinned by shared clinical trials, interoperable data infrastructures, and co-governed research agendas, thereby enhancing not only scientific rigor and reproducibility but also geopolitical and ethical equity in the advancement of precision oncology.

To summarize, INCT UroGen represents a new wave of Brazilian scientific leadership: anchored in excellence, propelled by strategic collaboration, and committed to the public good (Table-1). At a time when the global cancer burden is rising and disparities in care are widening, Brazil is uniquely positioned not only to contribute to global oncology but to lead, especially in contexts where innovation must be harmonized with equity and access.

**Table 1:** Science Embedded in SUS: What Makes INCT UroGen Unique.

Feature	Description
National Reach	Active nodes across all five Brazilian macroregions, enabling inclusive research participation and addressing regional disparities in cancer care.
Public Health Integration	Research outputs are designed to inform SUS clinical guidelines, policies, and delivery protocols—ensuring direct application in Brazil's public health system.
Cost-Effectiveness Validation	Emphasis on biomarker and technology validation for economic viability within SUS, promoting sustainable innovation.
Equity-Driven Mission	Projects are aligned with SUS's foundational principles of universality, equity, and integrality—bridging gaps in access to care.
Shared Infrastructure	Creation of interoperable biobanks, research databases, and multicenter trials fosters collaboration and reproducibility.
Capacity Building	Multicenter mentorship, graduate training, and fellowships develop human capital within public universities and health institutions.
Social Impact Metrics	Tracks research translation into guidelines, legislation, and care delivery, using Social Impact Factor (SIF) frameworks.
Public-Private-Public Synergy	Facilitates cross-sector partnerships while maintaining a mission-driven, public-first agenda anchored in SUS values.
International Collaboration	Strategic alliances with global cancer centers and funding agencies enable co-developed trials and knowledge exchange.
Long-Term Vision	Backed by stable programmatic funding (e.g., CNPq, FAPESP, FINEP, CAPES) and embedded in national scientific policy frameworks.
